# Evolution of Oculomics: From Naked Eye Observations to Artificial Intelligence over 100 Years

**DOI:** 10.1016/j.xops.2026.101079

**Published:** 2026-01-20

**Authors:** Anushka Irodi, Xiaofei Wang, Ya Xing Wang, Yih Chung Tham, Cecilia S. Lee, Carol Y. Cheung, Zhuoting Zhu, Andrzej Grzybowski, Tien Yin Wong

**Affiliations:** 1 School of Clinical Medicine, University of Cambridge, Cambridge, UK; 2 Key Laboratory for Biomechanics and Mechanobiology of Ministry of Education, Beijing Advanced Innovation Center for Biomedical Engineering, School of Biological Science and Medical Engineering, Beihang University, Beijing, China; 3 Beijing Visual Science and Translational Eye Research Institute (BERI), Eye Center of Beijing Tsinghua Changgung Hospital, School of Clinical Medicine, Tsinghua Medicine, Tsinghua University, Beijing, China; 4 Beijing Key Laboratory of Intelligent Diagnostic Technology and Devices for Major Blinding Eye Diseases, Tsinghua Medicine, Tsinghua University, Beijing, China; 5 Department of Ophthalmology, Yong Loo Lin School of Medicine, National University of Singapore, Singapore; 6 Centre for Innovation and Precision Eye Health, Yong Loo Lin School of Medicine, National University of Singapore and National University Health System, Singapore; 7 Singapore Eye Research Institute, Singapore National Eye Centre, Singapore; 8 Ophthalmology and Visual Science Academic Clinical Program (Eye ACP), Duke-NUS Medical School, Singapore, Singapore; 9 Department of Ophthalmology, University of Washington, Seattle, Washington; 10 Roger and Angie Karalis Johnson Retina Center, Seattle, Washington; 11 Department of Ophthalmology and Visual Sciences, The Chinese University of Hong Kong, Hong Kong, China; 12 Centre for Eye Research Australia, Ophthalmology, University of Melbourne, Melbourne, Victoria, Australia; 13 Department of Surgery (Ophthalmology), University of Melbourne, Melbourne, Victoria, Australia; 14 Department of Ophthalmology, University of Warmia and Mazury, Olsztyn, Poland; 15 Institute for Research in Ophthalmology, Foundation for Ophthalmology Development, Poznan, Poland; 16 Beijing Visual Science and Translational Eye Research Institute (BERI), Beijing Tsinghua Changgung Hospital Eye Center, School of Clinical Medicine, Tsinghua Medicine, Tsinghua University, Beijing, China; 17 School of Biomedical Engineering, Tsinghua Medicine, Tsinghua University, Beijing, China; 18 Singapore Eye Research Institute, Singapore National Eye Center, Singapore

**Keywords:** Oculomics, Retinal biomarkers, Systemic diseases, Artificial intelligence, Historical review

## Abstract

**Purpose:**

To provide a historical review tracing the evolution of oculomics, the study of ocular biomarkers of systemic disease, from early clinical observations to the emergence of advanced imaging and artificial intelligence (AI) technologies over the last century.

**Design:**

Narrative historical review.

**Subjects:**

Not applicable.

**Methods:**

A review of key historical and technological developments in ocular examination, imaging, and data analysis that have shaped the modern field of oculomics.

**Main Outcome Measures:**

Not applicable.

**Results:**

The central concept underlying oculomics—the eye being a uniquely accessible site for detecting systemic disease—has long been recognized in clinical practice. Over the past century, advances in imaging and computational methods have enabled increasingly precise quantitative measurements of retinal biomarkers able to detect cardiovascular, neurodegenerative, and metabolic diseases. Recent integration of AI-based image analysis has further expanded research and diagnostic potential.

**Conclusions:**

Oculomics, while recently formalized as a defined field, represents the convergence of historical clinical insights with modern technological innovation. By situating current advances within a broader historical context, this review highlights the way in which the role of the eye in systemic disease detection has evolved over the last century.

**Financial Disclosure(s):**

Proprietary or commercial disclosure may be found in the Footnotes and Disclosures at the end of this article.

The term “oculomics” was formally introduced by Wagner et al^1^ in 2020 to describe the convergence of ophthalmic imaging, big data analytics, and artificial intelligence (AI) in uncovering systemic information from the retina of the eye. While the name is new, the underlying concept is not. The idea that the eye serves as a mirror of internal health has deep historical roots, predating modern imaging by more than a century, to times when physicians still relied on naked-eye examination and early ophthalmoscopes to recognize telltale signs of disease. Classic examples include the copper-colored Kayser–Fleischer rings of Wilson disease, flame-shaped hemorrhages in hypertensive retinopathy, and cotton-wool spots signaling microvascular ischemia in diabetes.^2^, ^3^, ^4^ However, this concept has been reshaped and strengthened by accelerating technological progress over the years—from early ophthalmoscopic examination and hand-drawn retinal sketches, we now have high-resolution imaging modalities such as OCT, digital fundus photography, and increasingly sophisticated AI-driven image analysis. These tools have allowed for quantification of retinal microvascular and neural changes that can signal systemic pathology, sometimes before clinical symptoms are seen elsewhere in the body.

In this review, we define oculomics broadly, encompassing both traditional ophthalmoscopic signs and modern data-driven imaging biomarkers, framing the latter as the most recent phase in a longer historical continuum. By highlighting landmark discoveries and shifts in conceptual paradigms (Fig 1), we aim to provide a broad overview of the way in which modern-day oculomics, as first defined in 2020, has emerged to redefine the role of the eye from a passive reflector of disease to a powerful predictor of systemic health.

**Figure 1:**
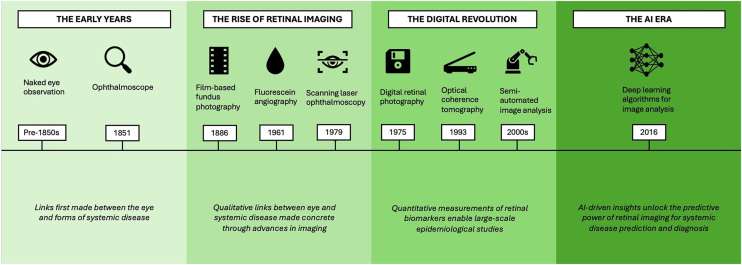
Key technological advances and evolving concepts in oculomics over time. AI = artificial intelligence.

## The Early Years: Naked Eye Observations and Fundoscopy (1830s–1950s)

One of the earliest recorded associations between ocular signs and systemic disease can be traced back to Richard Bright in 1836, who described vision loss in patients with albuminuria.^5^ He linked visual changes to underlying kidney disease, laying the foundation for the concept of the eye as a window into microvascular pathology. At the time, the study of ocular manifestations relied heavily on external observation of the anterior eye or patient-reported symptoms, as no direct visualization techniques were available.

Yet, curiosity about visualizing the posterior segment of the eye had long predated this. In 1703, Jean Méry observed the glow of a cat’s eye when submerged in water, allowing easier visualization of retinal vessels.^6^ A century later, in 1823, Jan Evangelista Purkinje used a myopic lens and candlelight to examine the fundus of dogs and humans, describing the red reflex and internal reflections from the eye—though his findings, published in Latin, went largely unnoticed.^7^ Ernst Wilhelm von Brücke later made similar observations, noting the reddish illumination of the pupil under specific lighting, and came close to conceptualizing a working device.^8^ A final notable claim to precedence came from the mathematician and inventor Charles Babbage, in 1847. He devised an early ophthalmoscopic instrument using a partially silvered mirror to reflect light into the eye while allowing the examiner to look through its center. Although it lacked the necessary optical correction to produce a clear retinal image, it anticipated several key principles of ophthalmoscopy.^9^ It was ultimately Hermann von Helmholtz, in 1851, who refined and popularized these ideas into a reliable, practical instrument: the ophthalmoscope. By introducing both illumination and optical correction into a single device, Helmholtz made it possible to directly visualize the living human retina for the first time.^10^ While numerous ophthalmologists went on to develop variations of the device—with more than a hundred different models displayed at a meeting hosted by ophthalmologists Friedenwald and Wood in Atlantic City, New Jersey, on the 50th anniversary of the invention^11^—Helmholtz’s original design established the 3 fundamental principles that remain essential to the ophthalmoscope's function: a light source, a reflective surface to project light into the eye, and an optical system to correct the observer's view of the retina.^12^ This breakthrough provided unprecedented direct visualization of the eye's internal structures.

Building on this, in 1866, Polish ophthalmologist Xavier Galezowski (1832–1907) emerged as a pioneer in using fundus examinations to detect central nervous system disorders. He published one of the early textbooks on this subject and coined the term, “cerebroscopy,” to describe this method of examination.^13^ Throughout the late 19th and early 20th centuries, ocular findings were increasingly reported in conjunction with systemic diseases. In 1872, Moritz Roth described Roth spots—retinal hemorrhages with white centers—in patients with infective endocarditis.^14^ Kayser–Fleischer rings, first described by Bernhard Kayser in 1902 and later by Bruno Fleischer in 1903, became a hallmark diagnostic feature of Wilson disease,^2^^,^^3^ reflecting copper deposition in Descemet membrane of the cornea. Henrik Sjögren described keratoconjunctivitis sicca as part of a syndrome with xerostomia in 1933,^15^ and Benediktos Adamantiades and Hulusi Behçet identified the triad of uveitis, oral ulcers, and genital ulcers in the same decade.^16^

**Figure 2:**
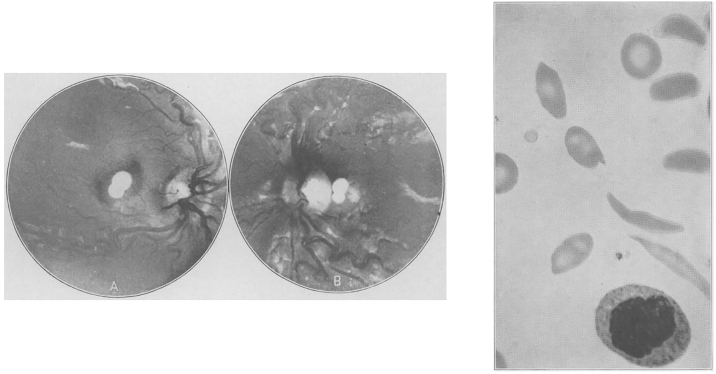
Retinal abnormalities and sickled erythrocytes from Harden’s 1937 report, one of the earliest documented descriptions of sickle cell retinopathy.

Causative definitive links between systemic disorders and their manifestations in the eye also began to take greater shape during this period. The first recorded case of sickle cell retinopathy was described by Cook in 1930.^17^ This was followed by Harden in 1937, who observed dilated and tortuous retinal vessels in patients with sickle cell disease^18^ (Fig 2). Pathophysiological links—vitreous hemorrhage, neovascularization, and retinal infarction due to sickled cells—began to be made a decade later in the 1940s, offering mechanistic explanations for the observed correlation.^19^, ^20^, ^21^ Certain associations, however, were more contentious. Eduard Jäger used the ophthalmoscope to describe diabetic macular changes, characterized as early as 1856,^22^ compiling one of the first atlases of ocular fundus paintings, painstakingly drawn over an average of 20 clinical sessions per patient.^23^ However, these observations were disputed by Albrecht von Graefe, a prominent ophthalmologist of the time, who argued that no definitive link between these changes and diabetes had been established.^24^ The debate persisted until 1872, when Edward Nettleship provided histopathological evidence of “cystoid macular degeneration” in patients with diabetes, finally settling the controversy.^25^

**Figure 3:**
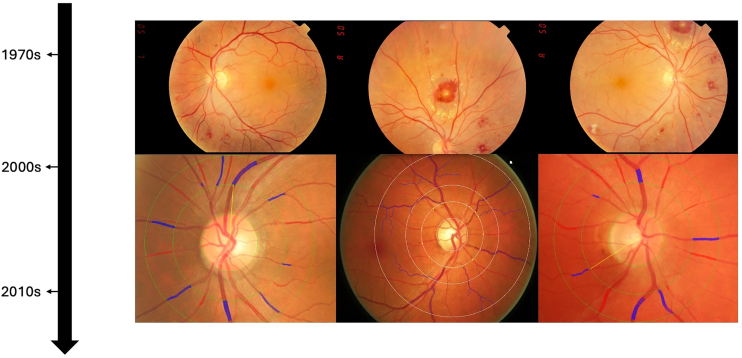
Evolution of hypertensive retinopathy retinal images over time from simple fundal observation to advanced imaging techniques.

One of the most significant contributions to early oculomics was the identification of retinal vascular signs in hypertension. In 1898, Marcus Gunn described a range of retinal vascular changes including arteriolar narrowing, arteriovenous nicking, retinal hemorrhages and exudates, and cotton wool spots in patients with kidney disease and hypertension^26^^,^^27^ (Fig 3). His work provided a clear correlation between systemic hypertension and ocular changes in a systematic and measurable way, a major breakthrough at the time. This work paved the way for a landmark study by Keith and Wagener in 1939, which standardized the grading of hypertensive retinopathy and demonstrated that the severity of these retinal vascular changes correlated with cardiovascular mortality.^4^ Despite the indisputable importance of this study, its methodology—relying on the subjective quantification of retinal vascular changes—demonstrated poor reproducibility.^28^ Intraobserver and interobserver variabilities, when the method was later applied to patients with mild to moderate hypertension, ranged from 20% to 42% to 10% to 30%, respectively.^29^ Consequently, alternative markers of hypertension-related end-organ damage, such as left ventricular hypertrophy and microalbuminuria, garnered greater attention in the following decades.^30^ Nevertheless, the early years of oculomics, driven by direct observation and marked by the invention of the ophthalmoscope, laid the foundations for the first shift toward structured, quantifiable assessment of ocular signs in systemic disease.

## The Rise of Retinal Imaging (1880s–2000s)

The ability to systematically capture and measure retinal features emerged with the development of fundus photography. Shortly after the invention of the ophthalmoscope, in the 1860s, Henry Noyes of New York and A.M. Rosebrugh of Toronto both constructed fundus cameras and attempted fundus photography on animals.^31^^,^^32^ The first *in vivo* photograph of the human fundus was published by Jackman and Webster in 1886,^33^ with refinements by Howe soon after.^34^ A persistent challenge in early fundus photography was achieving adequate illumination of the interior eye—a technical limitation that was eventually overcome with the invention of the stroboscopic flash by Edgerton in 1930, allowing consistent and clearer images.^35^

The next major imaging breakthrough came with the invention of fluorescein angiography by Novotny and Alvis in 1961, which allowed for dynamic, high-contrast visualization of the retinal vasculature.^36^ Two decades later, in 1979, Webb et al introduced scanning laser ophthalmology, a technique that employed laser light to illuminate small areas of the retina sequentially, generating high-contrast confocal images with superior resolution.^37^ This innovation enabled more detailed assessment of the optic nerve head and retinal nerve fiber layer (RNFL), particularly in glaucoma and other neuro-ophthalmic conditions.^38^

With the support of imaging tools that allowed for reliable visualization and documentation, empirical links between retinal features and systemic disease gained traction. Retinal microvascular changes, such as arteriolar narrowing and vascular remodeling, were recognized as indicators of cardiovascular disease.^39^^,^^40^ Similarly, optic nerve degeneration and RNFL thinning were observed in neurodegenerative conditions such as Alzheimer disease.^41^^,^^42^ Post-mortem studies^43^^,^^44^ further reinforced these connections, demonstrating links between optic nerve degeneration and dementia as well as retinal microvascular abnormalities and stroke. These findings highlighted how anatomically distinct regions of the eye—such as the retinal vasculature and optic nerve head—could serve as biomarkers for different systemic diseases, reflecting their shared embryological origins and physiological parallels with the cardiovascular and central nervous systems, respectively. The ability to measure parameters such as retinal vascular caliber quantitatively from fundus photographs also provided a new approach to assessing risks for disease development. Studies utilizing film-based fundus photography identified early vascular changes in diabetes, sometimes preceding clinical signs of diabetic retinopathy.^45^ These findings highlighted the burgeoning potential of retinal imaging not only as a diagnostic tool for early disease states but also as a means of disease prediction.

A persistent challenge throughout this early period of traditional film-based fundus photography was the lack of repeatability and standardization. Measurements were highly susceptible to variability arising from differences in image acquisition, manual annotation methods, and intraobserver and interobserver analyses,^46^ limiting reproducibility across studies. In addition, the absence of widely accepted protocols and calibrated imaging tools further reduced the clinical applicability of findings. Despite this, the rise of retinal imaging represented a transformative period, shifting the field from qualitative observations to data-driven analysis.

## The Digital Revolution: Quantitative Oculomics (1990s–2015)

The shift from film to digital retinal imaging in the late 20th century marked a critical step in the evolution of the field. It was driven by 2 key breakthroughs in retinal imaging hardware: digital fundus photography and OCT.

The invention of the first digital camera by Steven Sasson at Eastman Kodak in 1975 marked a broader transition from analog to digital imaging across multiple domains, including medicine.^47^ By the 1990s, digital fundus photography had become increasingly integrated into ophthalmic practice, offering a distinct advantage over conventional film-based techniques. Early validation studies demonstrated that digital fundus photography showed superior sensitivity in detecting retinal abnormalities compared to mydriatic ophthalmoscopy.^48^^,^^49^ Importantly, digital images enabled quantitative analysis of retinal features, shifting the paradigm from subjective qualitative assessments to objective, reproducible measurements.

Large-scale epidemiological studies such as the Atherosclerosis Risk in Communities Study, Cardiovascular Health Study, Multi-Ethnic Study of Atherosclerosis, Beaver Dam Eye Study, Blue Mountains Eye Study, and Rotterdam Study adopted digital retinal photography to collectively demonstrate that subtle retinal vessel changes, such as narrower arteriolar caliber and wider venular caliber, could predict the future risk of hypertension, stroke, coronary heart disease, and heart failure.^50^, ^51^, ^52^, ^53^, ^54^, ^55^ In parallel, the introduction of OCT in the early 1990s allowed for *in vivo* cross-sectional imaging of the retina with micrometer-scale resolution. This enabled detailed assessment of the RNFL, macula, and optic nerve head. Its applications were soon extended to systemic neurological conditions such as Alzheimer disease and multiple sclerosis,^56^^,^^57^ with longitudinal studies linking RNFL thinning and macular volume loss to cognitive decline, dementia, and cerebral small vessel disease.^58^, ^59^, ^60^ Over time, OCT proved useful for evaluating disease progression and serving as a biological marker of neuroaxonal injury across a wide range of conditions including Parkinson disease, Huntington disease, Friedreich ataxia, intracranial hypertension, migraine, neurosarcoidosis, schizophrenia, obstructive sleep apnea-hypopnea syndrome, bipolar disorder, and several rare neurological syndromes.^61^

The increased availability of digital imaging data spurred the development of software tools for retinal image analysis. Early semi-automated systems such as interactive vessel analysis and Singapore I Vessel Assessment enabled rapid, objective, and standardized quantitative measurements of features such as retinal vascular caliber.^50^^,^^62^ Other retinal “architectural” features such as fractal dimension, tortuosity, bifurcation optimality, and branching angles could now be assessed through network analyses^63^ (Fig 3). These metrics were used to study not only cardiovascular outcomes but also conditions as varied as diabetes, schizophrenia, and cerebral small vessel disease.^64^, ^65^, ^66^ These studies firmly established the conceptual role of the retina as a noninvasive surrogate for systemic health. Table 1 summarizes the key milestones and conceptual shifts of oculomics in cardiovascular, neurological, and other systemic domains over time. While numerous reviews have critically evaluated the performance and accuracy of individual studies over time in these domains,^1^^,^^58^^,^^70^, ^71^, ^72^^,^^75^, ^76^, ^77^, ^78^ here we provide a broad historical overview of developments, noting that most findings have remained experimental and have not yet entered routine clinical practice.

**Table 1:** Key Milestones and Conceptual Shifts in Oculomics across Systemic Disease Domains

Time Period	Key Milestones	Conceptual Shift
Cardiovascular		
1830s–1850s	Richard Bright links retinal changes to kidney disease.^5^	First recognition that retinal changes reflect systemic vascular pathology.
1870s–1900s	Roth spots linked to infective endocarditis;^14^ Marcus Gunn describes hypertensive retinopathy.^26^^,^^27^	Retinal vascular abnormalities are recognized as indicators of systemic vascular disease.
1930s–1950s	Keith*–*Wagener*–*Barker classification standardizes qualitative grading of hypertensive retinopathy.^4^	Correlates severity of retinal vascular changes with cardiovascular mortality.
1960s–1980s	Early quantitative studies of arteriolar narrowing; retinal signs of CHD, stroke, HF observed.^39^^,^^40^	Quantitative retinal vascular changes are recognized as correlates of hypertension, CHD, stroke, and HF risk.
1990s–2010s	Large-scale epidemiological studies associate retinal vascular features with cardiovascular outcomes using reproducible semiautomated software.^50^, ^51^, ^52^, ^53^, ^54^, ^55^	Retinal imaging emerges as a quantitative tool for cardiovascular risk stratification.
2010s - present	AI models detect cardiovascular risk factors and predict risk of future events from retinal images.^67^, ^68^, ^69^	Experimental noninvasive prediction of future cardiovascular events.
Neurological		
1970s–1990s	Early imaging and histopathological studies showed optic nerve and retinal changes in Alzheimer disease and vascular changes in cerebrovascular disease.^41^, ^42^, ^43^, ^44^	Early recognition that the eye and brain share embryological and pathological mechanisms, supporting the concept of the retina as a “window to the brain.”
1990s–2010s	Introduction of OCT enables in vivo measurement of RNFL and macular volume, with studies linking thinning to cognitive decline, dementia, and small vessel disease.^56^, ^57^, ^58^, ^59^, ^60^, ^61^	Retinal structure emerges as a noninvasive biomarker of neurodegeneration and cerebrovascular pathology.
2010s-present	AI models explore prediction of Alzheimer, cognitive decline, and other neurological outcomes from retinal images.^70^, ^71^, ^72^	Experimental application of oculomics to early detection of neurological disease, extending beyond structural markers to predictive models.
Other		
1850s–1950s	A wide variety of systemic disease signs manifesting in the eye recognized, e.g., corneal deposition in Wilson disease,^2^^,^^3^ diabetic retinopathy^4^ and diabetic macular edema^25^ and sickle cell retinopathy changes^18^—among many others.	Concept of the eye as a surrogate marker for systemic health emerges.
1990s–2010s	Retinal features begin to be associated with a wide variety of systemic diseases—including predictions of diabetic retinopathy before clinical signs^45^ and psychiatric disease such as schizophrenia.^65^	Retinal biomarkers are proposed to be associated with conditions as varied as diabetic retinopathy and schizophrenia.
2010s–present	Experimental AI-based models are able to predict renal function (eGFR, creatinine), hemoglobin, and liver and thyroid biomarkers.^73^^,^^74^	Experimental extension of oculomics into multisystem health monitoring and AI-driven disease prediction beyond traditional cardiovascular and neurological domains.

AI = artificial intelligence; CHD = coronary heart disease; eGFR = estimated glomerular filtration rate; HF = heart failure; RNFL = retinal nerve fiber layer.

Several limitations to the work during this period persisted.^79^, ^80^, ^81^, ^82^ Firstly, retinal measurements were still affected by biological and environmental variability, image quality, and technical differences across platforms. Secondly, semi-automated systems often required manual correction, particularly for qualitative features such as focal narrowing or arteriovenous nicking, which were not reliably captured algorithmically. Finally, questions were raised about clinical utility: while retinal metrics could reproducibly predict disease risk at the population level, most were retrospective and experimental, and their added value beyond traditional risk factors for individual-level clinical decision-making was unclear.

These limitations highlighted the need for faster, more scalable, and fully automated tools. As digital repositories grew and machine learning methods matured, the stage was set for the next phase in oculomics.

## The AI Era: Oculomics as a Biomarker (2015–Present)

In the last decade, the explosion of AI, particularly deep learning, has introduced new approaches for analyzing retinal images at scale. Unlike conventional machine learning methods, which rely on manually engineered features, deep learning algorithms can autonomously learn intricate patterns from vast amounts of imaging data. This capability has enabled the development of predictive models for ocular and systemic disease, advancing the field once more.^1^^,^^75^

Early work by Abràmoff et al in 2010 demonstrated automated detection of diabetic retinopathy from retinal images, a milestone that ultimately contributed to the development of the first US Food and Drug Administration-approved autonomous AI diagnostic device in medicine. Subsequent experimental studies have applied AI-driven analysis of retinal images to not only detect established ophthalmic conditions, such as diabetic retinopathy and age-related macular degeneration, but also to predict a plethora of major systemic morbidities such as cardiovascular, renal, and even neurological disorders.^67^, ^68^, ^69^ Strikingly, experimental AI models have been able to infer demographic variables such as age and sex directly from retinal scans—features that even human experts cannot discern.^83^^,^^84^ These findings suggest that latent features, correlative to human health and unseen to human eyes, exist in retinal imaging. While these applications remain exploratory, these experimental results have garnered significant interest, with excitement surrounding the untapped potential of retinal imaging as a biomarker for broader health monitoring.^73^^,^^74^

A major driver of this progress has been the growth of large imaging datasets and high-resolution imaging modalities. The volume and complexity of these datasets have exceeded the limits of manual or semi-automated methods developed in the early digital era. Deep learning approaches offer a powerful, scalable alternative for analyzing such data, but alongside their strengths, also raise their own set of challenges, summarized in Table 2. Key among these are interpretability and explainability: deep learning models often lack transparency in how predictions are derived, limiting clinical trust and regulatory acceptance.^85^ While explainable AI techniques are under development to address these concerns,^89^ the integration of such models into clinical workflows remains limited. Other major challenges to clinical implementation yet to be tackled include the lack of generalizability across diverse populations, the need for large-scale prospective trials of models for clinical validation, and clear plans for integration into clinical workflows.^85^^,^^86^ These challenges hinder translation by reducing confidence in model outputs and their reliability, alongside presenting practical barriers to safe, unbiased, and reproducible deployment in clinical settings.

**Table 2:** A Broad Overview of the Key Strengths and Challenges of AI in Oculomics

AI in Oculomics	Strengths	Challenges
Automated accurate quantitative analysis	Rapid objective accurate measurements of retinal features that reduce manual labor and improve reproducibility.^72^	Interpretability and explainability: complex models are “black boxes” with clinicians unable to audit which retinal features are driving predictions.^85^
Detection of subtle patterns	Experimental capability to identify even features not visible to human experts and draw accurate systemic disease or demographic variable associations.^83^^,^^84^	Lack of generalizability and domain shift: training on limited demographically nondiverse datasets can reduce generalizability of findings and introduce bias.^86^
Prediction & risk stratification	Models are able to estimate disease risk and predict future outcomes with a high degree of accuracy.^68^^,^^69^	Interpretability and explainability: population-level metrics (AUC, sensitivity, and specificity) do not guarantee reliability for individual patients, and the lack of interpretability can make tools difficult to trust and implement by users.^85^
Scalability & accessibility	Models can handle large datasets efficiently, enabling population-level studies and automated screening. This holds potential to improve access in resource-limited settings.^86^	Clinical implementation is limited by difficulties in clear workflow integration, need for specialized infrastructure and clinician acceptance.^72^
Advanced AI & multimodal integration	Emerging foundation and vision-language models show promise toward integrating imaging with clinical data and generalizing across tasks.^87^^,^^88^	Multimodal integration further increases the complexity of standardization and clinical implementation challenges.
Clinical translation	Experimental models provide quantitative, reproducible metrics that have potential to accelerate early detection and stratification of systemic disease.	Lack of clinical validation: very few algorithms have undergone prospective or external clinical validation. Prospective trials are difficult to organize for AI tools due to the expertise required in labeling images, the long-term follow-up required, the specialized investigations for diagnosing systemic disease, the essential software infrastructure required, and the need for robust engagement from clinicians and participants. Moreover, wider concerns surrounding ethics, regulation, and data governance remain.^72^^,^^85^

AI = artificial intelligence; AUC = area under the curve.

More recently, foundation models and vision-language models have been explored for ophthalmic applications. These large-scale models are trained on diverse data sources and designed to generalize learning across tasks and domains,^87^^,^^88^ potentially improving transferability and reducing the need for task-specific datasets. Vision-language models, which integrate image and text inputs, offer a pathway to link retinal images with clinical records and diagnostic codes.^88^ While early experimental results are promising, their utility in routine clinical settings remains to be established (Table 2).

## Future Directions

Several priorities are likely to shape the next phase of oculomics^72^ and support translation of current experimental advances into actual clinical practice. Further advances in imaging technology, such as ultrahigh-resolution OCT, multimodal imaging, and emerging techniques like adaptive optics, will improve sensitivity to early disease states. Parallel progress of AI models will need to focus on strategies that directly tackle the challenges hindering clinical implementation (Table 2). These include incorporating long-term clinical outcomes data into model development—such as incident cardiovascular events identified through longitudinal health-record linkage—improving explainability, and ensuring robust validation across diverse populations and clinical settings^90^ (Table 2). Evidence-based regulatory frameworks and guidelines will need to evolve to address challenges related to algorithm transparency, data privacy, equity, and practical clinical interpretation and implementation. Further research and consensus will be required to define the optimal role of these tools within routine care, and embedding them into interoperable clinical workflows, with appropriate clinician oversight and prospective evaluation, will be essential for successful translation. Integrating oculomics with multi-omics data such as genomics and proteomics may further support the discovery of novel biomarkers relevant to systemic diseases.^91^ Finally, interdisciplinary collaboration across specialties will be essential, as the applications of oculomics begin to span beyond ophthalmology into cardiology, neurology, and metabolic medicine.

## Conclusion

Just as Helmholtz’s ophthalmoscope revolutionized ophthalmology in the 19th century, the advent of AI and large-scale data is beginning to transform how we interpret the eye in the 21st century. Rather than representing a sudden shift, however, it is clear that the evolution of modern-day oculomics has been built on a centuries-old enduring concept: that the eye offers a uniquely accessible view into systemic health. Each generation of tools, from early drawings and film photography to OCT and AI analysis, has advanced our ability to visualize, quantify, and interpret retinal features with greater clarity and confidence. While recent advances in AI and large-scale imaging have begun to accelerate discovery and improve precision, they represent the latest step in a much longer historical journey of clinical observation, epidemiological research, and technological refinement.
